# Gender-Related Effect of Sodium Dichloroacetate on the Number of Hassall's Corpuscles and RNA NKCC1 Expression in Rat Thymus

**DOI:** 10.1155/2019/1602895

**Published:** 2019-04-24

**Authors:** Jūratė Stanevičiūtė, Milda Juknevičienė, Ingrida Balnytė, Angelija Valančiūtė, Vaiva Lesauskaitė, Julija Fadejeva, Rimantas Stakauskas, Donatas Stakišaitis

**Affiliations:** ^1^Department of Histology and Embryology, Medical Academy, Lithuanian University of Health Sciences, Kaunas, LT 44307, Lithuania; ^2^Institute of Cardiology of Lithuanian University of Health Sciences, Kaunas, LT 50009, Lithuania; ^3^Laboratory of Molecular Oncology, National Cancer Institute, LT 08660 Vilnius, Lithuania; ^4^Biological Research Center, Lithuanian University of Health Sciences, Kaunas, LT 47181, Lithuania

## Abstract

The aim was to investigate the effect of dichloroacetate (DCA) on thymus weight, Hassall's corpuscle number (HCs), and NKCC1 RNA expression in Wistar rats aged 4–5 weeks. They were investigated in the controls and DCA-treated gonad-intact and castrated males and females. The treatment lasted 4 weeks with DCA 200 mg/kg/day. At the end of the experiment, rat thymus was weighted, and its lobe was taken for the expression of NKCC1 RNA determined by the PCR method and of Hassall's corpuscles by immunohistochemistry. DCA caused a thymus weight decrease in DCA-treated gonad-intact rats of both genders as compared with their controls (p < 0.05), and no such impact was found in castrated DCA-treated males and females. DCA caused an increase of the HCs in gonad-intact males (p < 0.05), and no such increase in the DCA-treated gonad-intact females was found. There was gender-related difference in the HCs when comparing DCA-treated gonad-intact males and females: males showed significantly higher HCs (p < 0.05); no gender-related differences were found in the castrated DCA-treated groups. The* Slc12a2* gene RNA expression level was found to be significantly decreased only in gonad-intact and in castrated DCA-treated males. The authors discuss the gender-related DCA effects on the thymus.

## 1. Introduction

Sodium dichloroacetate (DCA) is an inhibitor of pyruvate dehydrogenase kinase (PDHK) [1]. DCA is absorbed from the gastrointestinal tract and transported across the cell membrane by the monocarboxylate transporter system and metabolized to monochloroacetate, glyoxylate, glycolate, oxalate, glycine, carbon dioxide, and chloride anion [2–4].

The DCA principal target is the pyruvate dehydrogenase complex (PDC). It inhibits all isoforms of PDHK, keeping PDC in the catalytically active form facilitating the oxidative removal of pyruvate. PDHK isoforms can phosphorylate E1*ɑ* (PDHA1), thus inactivating it; the mechanism for PDC inhibition is related with the posttranscriptional upregulation of one or more PDHK isoforms, leading to phosphorylation of the E1*ɑ* subunit of PDC and maintaining the glycolytic profile of proliferating cells [1, 5, 6].

The PDHA1 gene is located on the X chromosome, and such gene location has different consequences for males and females with PDHA1 congenital deficiency; gender-related clinical problems depend mainly on the residual PDHA1 enzyme activity; PDC congenital or acquired deficiency may be a cause of lactic acidosis [7–9].

Pyruvate dehydrogenase is present in normal and in cancer tissues, and the PDC/PDHK axis has been suggested as a specific target in cancer treatment [10, 11]. DCA has been employed for indication in the chronic treatment seeking to decrease the blood lactate acid level in congenital lactic acidosis [12] or to inhibit the anaerobic glycolysis which renders various cancer cells resistant to apoptosis induction [13]. DCA induces apoptosis, cell cycle arrest, reverses the Warburg effect in cancer cells [11, 14, 15], increases apoptosis via the intrinsic mitochondrial pathway due to the high reactive oxygen species causing mitochondrial depolarization, decreases the ATP production, and effectively kills tumor cells [16, 17].

Thymus could be a valuable model for investigating the effect of medicinal products on thymocyte proliferation. Following castration, hyperplasia of thymus and gender-related increase in the Hassall's corpuscles number (HCs) in thymus appear in rats [18]. HCs are related to the loss of apoptotic thymocytes and maturation of developing thymocytes [19]. The DCA treatment shows efficacy in reducing proliferation in highly proliferating normal cells, e.g., rat thymocytes; it has been related with thymus weight loss in gonad-intact DCA-treated male rats and a decrease of the thymocyte number in the G_0_–G_1_ phase as well as thymocyte accumulation in the G_2_–M phase, but no significant effect was found on thymus weight or thymocyte cell cycle in DCA-treated castrated males, indicating that DCA works synergistically with gonad hormones in males [20].

Recently, we reported inhibition of Na^+^/K^+^/2Cl^−^ cotransporter (NKCC) by DCA in male rats: (1) a single dose significantly increased 24-hour urinary output as well as Cl^−^, Na^+^, K^+^, Ca^2+^, and Mg^2+^ excretion; (2) changes following 4-week treatment included increase in the size of the Henle loop's thick ascending limb's epithelial cells (an effect related to NKCC2 inhibition) and (3) significant decrease in RNA NKCC1 expression in thymus of male rats [21]. The intracellular chloride level in rat thymocytes partially is regulated by chloride influx via the NKCC1 functional activity [22, 23]. The NKCC1 gene also is known as a solute carrier family 12, member 2 (*Slc12a2*). This gene was found to be broadly expressed in thymus [24, 25]. NKCC1 participates in cell proliferation and tumorigenesis processes [26].

J. A. Clayton (2014) noted that female and male cells differ in their response to chemical agents but preclinical research often neglects association of medicines effects with gender and sex hormones [27]. We did not find any study on gender-related effects of DCA on thymus in literature. The article presents data on gender-related effect of repeated dosing of DCA on thymus of normal and castrated rats of both genders including its weight, Hassall's corpuscle number, and NKCC1 RNA expression.

## 2. Materials and Methods

### 2.1. Study Design

The effect of the DCA treatment on the thymus was investigated in the following 8 groups of age-matched Wistar rats of both genders: gonad-intact and castrated male and female controls and in respective male and female DCA-treated groups.

The permission was obtained from the State Food and Veterinary Service of Lithuania to use experimental animals for research (2015-05-18 No. G2-28). The animals were purchased from the Animals Facility of the Veterinary Academy at the Lithuanian University of Health Sciences (Kaunas, Lithuania). The experiment was carried out at the Animal Research Center at the Lithuanian University of Health Sciences (Kaunas, Lithuania). The animals were housed in standard colony cages with free access to food, in the conditions of constant temperature (21 ± 1°C), humidity, and the light /dark cycle (12 h / 12 h). A commercial pellet diet was provided* ad libitum*. The experiments were performed in compliance with the relevant laws and institutional guidelines for animal care in order to avoid any unnecessary animal distress.

For the experiment, 4–5-week aged Wistar rats were selected with the same animal number (n = 6) in the groups; there was no difference in rat weight among the formed groups. In the animal groups selected for castration, the male orchidectomy and female ovariectomy operations were performed. The castration was performed at the age of 28 ± 2 days (in the peripubertal period of animals). The accommodation period after the castration was one week. After the accommodation period, the treatment of gonad-intact and castrated animals was started. At the end of the experiment, one castrated DCA-treated female was eliminated from the study due to a fistula formed after the operation and significant weight loss.

Treatment with DCA aqueous solutions (200 mg/kg/day) in drinking water was used. The only source of drinking was the DCA solution for treated groups, and fresh tap water was provided for the control groups; DCA solution and water were offered to animals* ad libitum. *The treatment duration was 4 weeks. The DCA dosage, administration in drinking water, and thymus preparation methods were described as previously [20].

### 2.2. The Thymus Preparation

Completing the experiment, the animals were killed in a 70% CO_2_ camera. To minimize the thymus contamination with red blood cells, the* carotid* arteries and the aorta were cut and the animals exsanguinated. Upon killing the animals, their thymus was harvested and the contaminating blood was removed by rinsing with RPMI-1640 (Biological Industries, Israel). The weight of the thymus was evaluated, and the left rat thymus lobe samples of the study groups after thymus surrounding connective tissue were removed and the thymus lobe was stored in the RNA*later*RNA stabilization reagent (Qiagen, Germany) at -80°C until the further RNA extraction and RNA analysis. The right lobe of the thymus was taken for the histomorphometric evaluation.

### 2.3. The HCs Determination by Histology

The right lobe of the thymus was fixed in 10% neutral-buffered formalin, embedded in paraffin, sectioned in 3 *μ*m sections, and stained with hematoxylin and eosin (H-E). For immunohistochemical examination, slices were placed on poly-L-lysinecoated glass slides. After deparafinization in xylene and rehydration, the sections were pretreated with the antigen-retrieval solution (0.01 mol/L of citrate buffer, pH 6) in a pressure-cooker and then incubated with cytokeratin monoclonal antibodies (clone 34 E12, dilution 1:50, Dako A/S, Denmark) for the identification of high molecular weight cytokeratins (HMW CK). Antibodies detection was performed with the EnVisionPlus-HRP kit (Dako, Denmark). Sections were counter-stained in weak Mayer's hematoxylin. The histological and immunohistochemical evaluation of the samples was performed with an OLYMPUS BX40F4 (Olympus Opticae co. LTD, Japan) microscope using the CellSens Dimention 1.9 Digital Imaging Software for Research Applications (Olympus Corporation of the Americas, USA). Histological sections were selected from the middle portion of the right thymus lobe. The total area of the thymus lobe and the area of the medulla were counted. The presence of Hassall's corpuscules, which are heterocellular, consisting of thymic epithelial cells (major cellular component), macrophages, interdigitating dendritic cells, myoid cells, and, occasionally, mast cells and lymphocytes [28] was evaluated and were counted in the medulla. The data are presented as the median per mm^2^ of thymus medulla in each group. The methodology of the HCs histological examination was as described previously [18].

### 2.4. Extraction of RNA from the Thymus

Rat thymus samples of all study groups were stored in RNA*later*RNA stabilization reagent (Qiagen, Germany) at -80°C until further RNA extraction. The frozen tissue was ground in liquid nitrogen. Total RNA was extracted using the TRIzol™ Plus RNA Purification Kit (Life Technologies, USA) according to the manufacturer's instruction. The RNA quality was assessed using a NanoDrop2000 spectrophotometer (Thermo Scientific, USA) using the A260/280 ratio. The extracted RNA samples were stored at -80°C until further analysis. The methodology of RNA extraction and the evaluation of the NKCC1 expression in thymus were used analogically as described [21].

### 2.5. Determination of the NKCC1 Expression in Thymus

RNA expression assay was performed for* Slc12a2* (Rn00582505_m1) and* Glpdh* (Rn01775763_g1) genes. High-Capacity cDNA Reverse Transcription Kit with RNase Inhibitor (Applied Biosystems, USA) was used for reverse transcription reaction in 20 *μ*l reaction volume containing 50 ng of total RNA incubated at 25°C for 10 min, transcripted at 37°C for 120 min, and terminated by heating at 85°C for 5 min using Biometra TAdvanced thermocycler (Analytik Jena AG, Germany). The synthesized cDNA was stored at 4°C until use or at -20°C for longer time. The Real-time PCR was run in triplicate with 4 *μ*l of cDNA template in a 20 *μ*l reaction volume (10 *μ*l of TaqMan Universal Master Mix II, no UNG (Applied Biosystems, USA), 1 *μ*l of TaqMan Gene Expression Assay 20x (Applied Biosystems, USA), and 5 *μ*l of Nuclease-Free Water (Invitrogen, USA) with the program running at 95°C for 10 min, followed by 40 cycles of 95°C for 15 s and 60°C for 1 min. the reaction was performed using an Applied Biosystems 7900 Fast Real-Time PCR System (Applied Biosystems, USA).

### 2.6. Statistical Analysis

The statistical analysis was performed by using the Statistical Package for the Social Sciences, version 22.0 for Windows (IBM SPSS Statistics V22.0, USA). The normality assumption was verified by the Kolmogorov–Smirnov test. The animal weight data are expressed as the mean ± SD values. The thymus weight data are presented as the mean and the 95% confidence interval (95% CI). When the normality assumptions are not met, data are expressed as a median and a range (minimum and maximum values). Differences between two independent groups were evaluated using the nonparametric the Mann–Whitney* U* test. The one-way ANOVA analysis was used to determine significance among the groups, and post hoc tests with Fisher's least significant difference were used for comparison among the individual groups. To investigate the* NKCC1* (*Slc12a2*) RNA expression changes in the DCA-treated group, the threshold cycle (CT) values were normalized with the control* Glpdh* gene; for the gene expression study, the delta delta threshold cycle (2^-ΔΔCT^) method was used to calculate the expression ratio between the DCA-treated (test) and control conditions of the target gene as compared with the reference gene. The Spearman's rank correlation coefficient (*r*) was used to assess the relationship among thymus weight, HCs and ∆CT. The Fisher's transformation which changes* r* to a Z-score and the Steiger's Z-test for comparison of correlations (*z*) within the population were used. Differences at the value of* p* < 0.05 were considered significant.

## 3. Results

### 3.1. The Relationship of the Rat Thymus Weight with the Rat Weight in the Study Groups

The data of rat thymus weight of the study groups after the treatment and their control groups are summarized in Table 1. No difference of the initial rat body weight was found between the control female and male groups (respectively, 77.33 ± 15.28 and 70.33 ± 6.81, p > 0.05) and the other study groups formed for castration and treatment did not differ according to body weight (p > 0.05).

At the end of the experiment, the gonad-intact males had a significantly higher body weight as compared with the gonad-intact female control (255.89 ± 48.30 and 191.62 ± 23.64, p < 0.007), and there was no significant rat body weight difference when comparing the gonad-intact male control and the gonad-intact DCA-treated males (p > 0.05); no difference was found between the respective female groups (p > 0.05).

The castrated DCA-treated rat groups of both genders showed a statistically significant body weight decrease as compared with the castrated control groups: the body weight of castrated DCA-treated males was by 15.07% lower than that of the control (245.12 ± 17.53 g and 208.17 ± 22.78 g; p < 0.011) and by 16.76% lower in the DCA-treated females as compared with their control (229.80 ± 20.84 g and 191.28 ± 24.50 g; p < 0.02).

### 3.2. The Data on the Effect of Castration and Treatment on the Rat Thymus Weight

**Figure 1:**
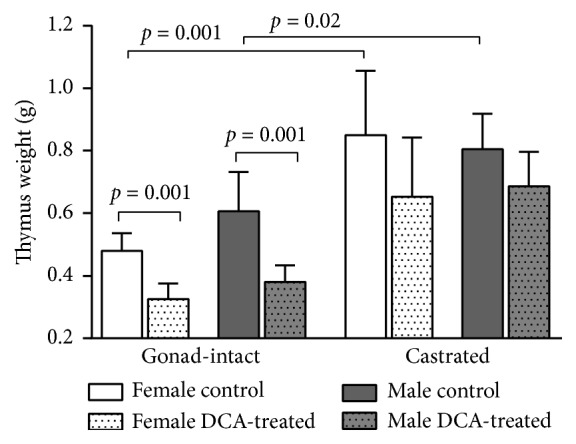
DCA treatment impact on rat thymus weight in gonad-intact and castrated rat groups of both genders. Data presented as a mean and 95% confidence interval.

The data on the effect of DCA treatment on thymus weight are shown in Table 1 and Figure 1.

A comparison of the gonad-intact control and the castrated control of both gender groups indicated a statistically significant thymus weight increase in castrated males (*p* = 0.02) and females (*p* = 0.001) control groups, because castration is related with thymus hyperplasia. There was no gender-related difference in animal thymus weight when comparing gonad-intact male and female as well as castrated rats (*p* > 0.05; Table 1).

The DCA treatment causes a significant thymus weight decrease in DCA-treated gonad-intact males (*p* = 0.001) and DCA-treated gonad-intact females (*p* = 0.001) as compared with their controls. No significant treatment impact was found in castrated DCA-treated male and female groups when comparing with their castrated controls (*p* > 0.05; Figure 1).

### 3.3. The Data on the Effect of Castration and Treatment on the Number of Hassall's Corpuscles in Rat Thymus

The HCs in the thymus of the studied groups are presented in Table 2.

**Figure 2:**
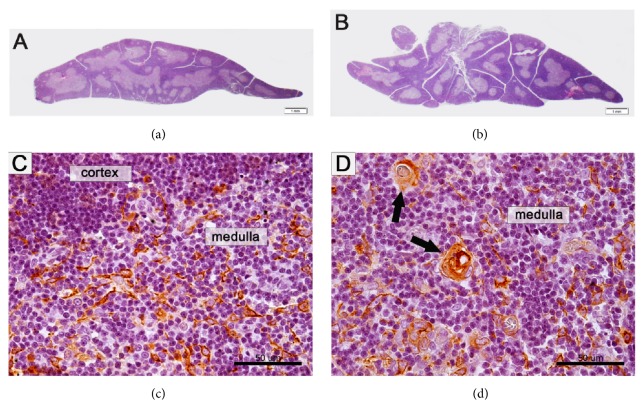
Morphological changes of rat thymus: a comparison of the male gonad-intact control and the male castrated control groups. (a) the male gonad-intact control group; (b) the male castrated control group (size of the male thymus increased after castration); (c) Hassall's corpuscles are not present in the male gonad-intact control rat thymus; thymic epithelial cells are positive for high molecular weight cytokeratins (clone 34*β*E12). Streptavidin-biotin-peroxidase, hematoxylin counterstain; (d) Hassall's corpuscles (arrows) are present in the medullar region of thymus in the male castrated control group and positive for high molecular weight cytokeratins. Scale bar: 1 mm (a and b), 50 *μ*m (c and d).

**Figure 3:**
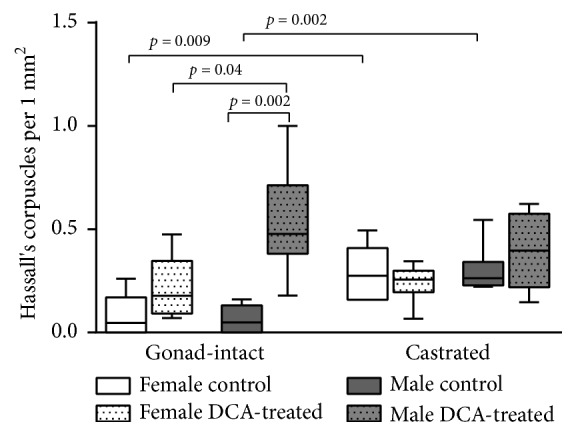
The Hassall's corpuscles number per mm^2^ in thymus of the gonad-intact and castrated rat groups of both genders. Data presented as a median and range (min–max).

The HCs appeared to be low in male and female thymus of gonad-intact control rat groups. There was a significant difference in the HCs between control gonad-intact and control castrated groups in males (*p* = 0.002) and females (*p* = 0.009). The treatment of rats with DCA caused a statistically significant increase of HCs in the male as compared with the gonad-intact control group (*p* = 0.002; Figure 2). The DCA treatment tends to increase the HCs in the female gonad-intact group, but no significant difference was found (*p* > 0.05). A significant difference in the HCs was found between DCA-treated gonad-intact males and females (*p* = 0.04), and males showed a significantly higher Hassall's corpuscle number. However, none of such differences were statistically significant in the castrated DCA-treated groups of both genders when comparing with the castrated control groups (*p* > 0.05; Figure 3).

**Figure 4:**
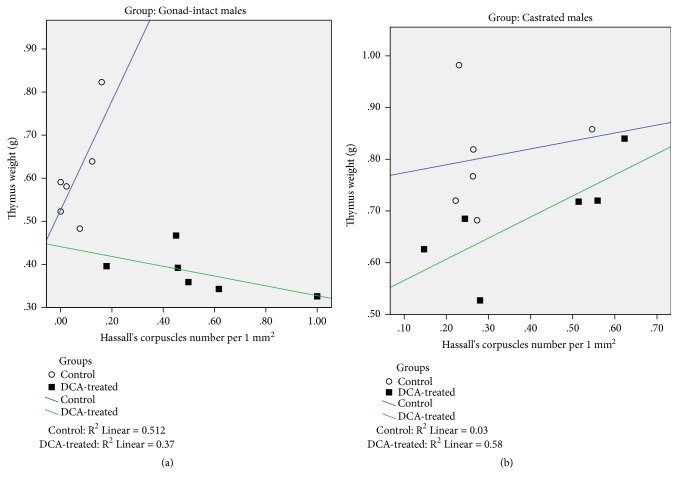
Correlation plots of the Hassall's corpuscles number per 1 mm^2^ in the thymus of gonad-intact (a) and castrated male (b) rat groups.

Comparison of the correlation between thymus weight and HCs of gonad-intact males and females controls with, respectively, correlations of DCA-treated groups shows that DCA treatment changes the relationship character: the correlations have positive direction in controls but after the repeated DCA dosage the correlation became negative; a comparison of correlation coefficients of gonad-intact DCA-treated males with their control revealed significant difference (p = 0.004). The correlation between thymus weight and HCs in gonad-intact and castrated DCA-treated male groups was significant (*p = *0.005 and* p *< 0.04, respectively, Figure 4). No significant data regarding the correlation between thymus weight and HCs in tested female groups were detected (Table 3).

### 3.4. The DCA Effect on RNA Expression of NKCC1 in Thymus

*Slc12a2* RNA expression in the thymus after normalization with* Glpdh* gene in analyzed rats groups is shown in Table 4.

**Figure 5:**
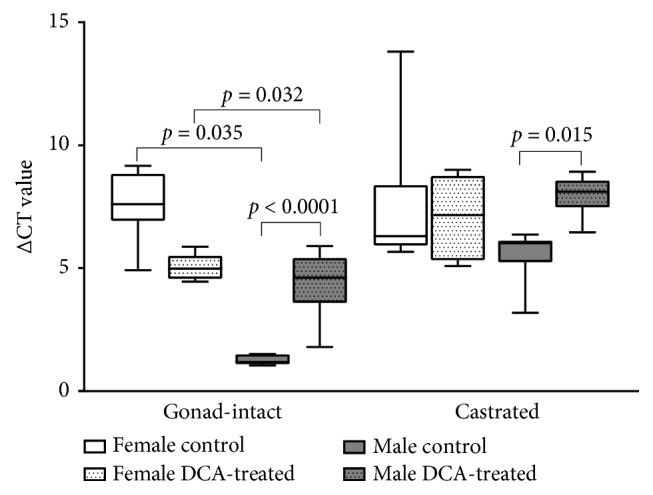
*Slc12a2* RNA levels in the rats after normalization with* Glpdh* gene. Delta threshold cycle (ΔCT) method was used for this analysis; the lower the ΔCT, the higher the expression (the horizontal bars represent the average, the minimal, and maximal values are shown with short horizontal lines).

Expression difference in the* Slc12a2* and* Glpdh* genes between the DCA-treated and the control groups is considered as the ∆CT value. The* Slc12a2* RNA levels in experimental groups after normalization with the* Glpdh* gene are shown in Figure 5. A significant difference was found between the ∆CT values of the gonad-intact male control and DCA-treated groups (p < 0.0001). In the castrated male group, the expression of the* Slc12a2* and* Glpdh* genes between the DCA-treated and the control groups was also significant (p = 0.015). The difference between the ΔCT of target and reference genes as expressed by the ΔΔCT is shown in Table 4. The RNA expression level (2^-ΔΔCT^) in the gonad-intact DCA-treated male was 0.103-fold change lower than in the control. This means the 90% downregulation of expression as the expression level is decreased by 90% to the level of 10% under control conditions. Also, there was a significant* Slc12a2* gene expression change in the castrated male DCA-treated group as compared with the control: its expression level was found to be decreased by 76% (Table 4). However, we found no statistical significance in the test RNA expression when comparing the control and the DCA-treated groups in both gonad-intact and castrated female rats (p > 0.05).

The correlation of thymus weight with ∆CT in the controls and in the DCA-treated gonad-intact and castrated groups of both genders was insignificant (p > 0.05); no significant differences comparing correlation coefficients of the control with the DCA-treated, respectively, groups were found (p > 0.05; Table 3).

## 4. Discussion

DCA has been employed as an investigational medicine for indication in the chronic treatment of conditions related to pathologically increased cell proliferation such as cancer and pulmonary hypertension [1, 10, 14]. The DCA principal site of action is to inhibit the PDHK, keeping the PDC in the unphosphorylated catalytically active form [5]. PDHK has been suggested as a specific target in proliferating cancer cells [13]. PDH is present in all tissues. Also, the DCA effect is related with a decrease of the blood lactate acid level [12]. Many aspects of the contribution of the DCA pharmacological mechanisms and their relationship with gender-related pathophysiological processes have not been investigated. PDH activity was higher in young female than in young male rats and decreased after ovariectomy but not after orchidectomy [29]. Estradiol treatment increased the expression of several PDH subunits [30]. Eight out of 11 genes evaluated for the PDC had higher expression levels in adult male hearts compared to females [31].

The rat thymus could be a valuable model for investigating the impact of a medicinal product on thymocyte proliferation. The study tested the hypothesis that the influence of DCA on thymus weight might be gender-related. It was based on the recent published data in which DCA has been shown to decrease thymus weight in gonad-intact male rats [20]. To exclude the influence of gonadal hormones on thymus involution, castrated male and female rats were studied also. We expected that changes might occur in HCs because they represent the stage of thymic epithelial cell differentiation [32, 33] and participate in removing the matured or apoptotic thymocytes [34, 35]. Both thymocytes and thymic epithelial cells possess androgen receptors [36].

A comparison of rat thymus weight of gonad-intact and castrated rats is related with a significant increase of thymus weight in castrated rats of both genders. Castration induces a thymus hyperplasia which is related with increased rat thymocyte proliferation [37]. Thymic involution is a complex process related with numerous molecular mechanisms, ageing, and gonad hormones included [38]. Androgens induce a decline of thymus weight in NZB mice, which results in the gender dimorphism of thymus weight between males and females [39]. In castrated female rats at the age of one month, the number of thymocytes and recent thymic emigrants in the peripheral blood was increased after a month as compared with the control animals [40]. The surgical and chemical castration of 12-month-old male rats caused regeneration of the atrophied thymus [41]. The Sprague–Dawley rat castration enhances thymic weight whilst gender hormones reduce the castration-induced thymus hypertrophy [42].

The study results show a decrease of thymus weight after four weeks of DCA treatment only in the gonad-intact males and females and no DCA treatment impact on thymus weight in the age-matched castrated DCA-treated male and female groups. These data indicate that the DCA acts on thymus weight synergistically with gonad hormones of both genders in gonad-intact males and females.

No relationship was determined between the thymus weight and the rat body weight in the tested DCA-treated groups of both genders despite the fact that castrated DCA-treated rat groups showed a significant body weight decrease; no rat body weight change was noted in the DCA-treated gonad-intact rat groups of both genders. A significant body weight decrease in DCA-treated castrated rats may indicate a change in the gonad-hormone-dependent DCA metabolism or a possible increased toxicity in the conditions of the reduced gonad hormone levels.

*The Effect of Castration and DCA Treatment on the HCs in Rat Thymus*. The study data indicate that the HCs appeared to be low in male and female thymus of gonad-intact control rat groups, and no gender-related difference was determined. These data confirm the literature data that HCs are poorly expressed in the thymus of rodents [43]. Tanaka et al. described HCs in spontaneous thymoma of 10-week-old Sprague–Dawley rats, and they were evaluated as medullar differentiation areas [44]. Rat castration is related with the increased HCs in female and male thymus [18].

The DCA treatment caused a significant increase of the HCs in the DCA-treated gonad-intact males as compared with their control, and no such increase in the DCA-treated gonad-intact females was found. There was gender-related difference in the HCs when comparing DCA-treated gonad-intact males and females: males showed significantly higher HCs, and no gender-related differences were found in the castrated DCA-treated groups when comparing the castrated control and castrated DCA-treated groups of both genders. The findings of DCA effect on thymus weight, on correlation between thymus weight and HCs may indicate that under the influence of DCA treatment the thymic epithelial cells undergo gender-related changes which could depend on the sex hormones.

In the thymus microenvironment, thymic epithelial cells are an important component supporting thymocyte development [45] which may be under the sex hormone control. When gonad-intact DCA-treated thymus diminishes in weight or increases in castrated animals, the increase in the HCs may have different pathophysiological mechanisms which can be related to the apoptotic thymocyte removal. Both thymic epithelial cells and thymocytes possess functional androgen receptors and can respond to testosterone [36]. The gonad-hormone-related mechanism of thymopoiesis is not clear; by using androgen-receptor knockout mice it was shown that thymic epithelial cells but not thymocytes or fibroblasts contribute to the determination of thymic cellularity [46]. The thymic epithelial cells in gonad-intact aged and in castrated male mice express similar sets of genes, and they are not altered after castration [47]. The mechanisms of the synergistic effect of DCA and testosterone on gonad-intact male thymus remain to be elucidated.

The study indicates some thymic epithelial cells to transform into HCs after reduction of gonad hormones by castration of both gender rats. When castrated male and female animals were treated with DCA, we noticed the further tendency of increase in HCs in the thymus of both sexes, and it was more pronounced in males. This supports the findings of Gray et al. that the thymic stroma is a dynamic population of thymocytes, and it may play a more direct role in the selection of thymocytes [48]. The study results suggest that different mechanisms are responsible for changes in thymopoiesis and thymus epithelial cells transformation into HCs under the DCA influence in gonad-intact and castrated animals. The DCA influences the thymopoiesis suppressing thymocyte proliferation, and this effect was gender-related and more pronounced in gonad-intact male rats; in contrast, in castrated rats after DCA treatment such DCA effect was not expressed.

*The DCA Effect on RNA Expression of NKCC1 in Thymus*. The existence of NKCC1 in rat thymocytes was the background to check the influence of DCA on its activity. The NKCC1 functional activity in rat thymocytes was found to be related with the chloride anion influx sensitive to the NKCC inhibitor furosemide, indicating that part of the chloride influx is mediated by NKCC1 [22, 23, 49].

The study shows that the DCA could target the NKCC1 protein where a gender-related difference of the DCA effect on the NKCC1 gene expression in rat thymus was determined. A significant difference was found between the ∆CT values (expression difference in the* Slc12a2* and* Glpdh* genes) in the DCA-treated gonad-intact and control males as well as in the castrated male groups. The* Slc12a2* gene RNA expression level (the 2^-ΔΔCT^ value) in the gonad-intact DCA-treated males was decreased by 90% under control conditions and significantly decreased (by 76%) in the castrated males. No changes in the tested NKCC1 RNA gene expression level were determined in the studied gonad-intact and castrated female DCA-treated groups as compared with the control of both gonad-intact and castrated female rats.

There are two NKCC isoforms: NKCC1 and NKCC2; the NKCC1 is distributed in various tissue types, among them in the thymus [24, 25] of different species [50]. NKCC1 mRNA levels were higher in males than in females on the day of birth, and the total NKCC1 protein levels were higher in the embryonic male than in female hypothalamus [51].

The DCA increases the reactive oxygen species generation [52]. The DCA produces time- and concentration-dependent increase in the superoxide anion and nitric oxide production in zebrafish [53]. The DCA effect on NKCC1 might be related with the oxidative stress because the NKCC activity in different cell types is regulated by oxidation and nitration, and the NKCC activity may depend on the levels of free oxidative radicals or nitric oxide donors; free oxidative radicals and protein tyrosine nitration can affect the NKCC structure and its function; the NKCC1 activity in endothelial cells was inhibited by the oxidant tertbutylhydroperoxide exposition [54, 55].

Young adult female mice have a lower oxidative stress and a higher pyruvate dehydrogenase complex activity as compared with young adult males, indicating that females may be better protected against the ROS damage. The depletion of steroids by ovariectomy potentially enhanced oxidative damage, whereas orchidectomy did not modify the oxidative stress parameters in mice [29]. The female rats showed a lower production of hydrogen peroxide in cardiac mitochondria as compared to males [56]. Increased formation of ROS inhibits the rat thymocyte proliferation [57].

The DCA treatment was reported to significantly reduce the thymus weight, and such changes are in concert with the DCA-induced increase of thymocyte percentage in the G_2_-M cycle phase and the reduced percentage of the G_0_-G_1_ phase [20]. The DCA-induced G_2_-M phase arrest in multiple myeloma cell lines induced by DCA was found also, where the described DCA effect supposed to be related with the oxidative stress caused by the DCA [17, 58]. Additionally, the increased reactive oxygen species generation in relationship with DCA treatment appears with a concomitant cellular shift from glycolysis to oxidative metabolism, resulting in an increased apoptosis [59]. The DCA causes Treg induction and Th17 suppression in the T-cell differentiation process which is dependent on the reactive oxygen species [60].

The induction of NKCC1 RNA expression suppression indicates that DCA may be important in regulating the intracellular chloride thymocyte concentration. The impact on the intracellular chloride concentration would have the antiproliferative effect [61, 62]. The NKCC1 plays an important role in cancer cell proliferation, apoptosis, invasion [62–64], has a potential role in cancer progression of tumors with a high NKCC1 expression, and is recognized as a cancer therapeutic target [26].

DCA acts as inhibitor of glutathione S-transferase-zeta1 (GST*ζ*). Elimination of DCA including glutathione- (GSH-) dependent oxygenation to glyoxylic acid mainly depends on GST*ζ*-catalyzed dechlorination by mitochondrial or cytosolic enzymes [65–67]. Regarding cytosolic GST*ζ*, the ^App^*K*_m_ for GSH obtained in female rats is 2.5–3.2-fold higher than males; the higher ^App^*K*_m_ of GSH is associated with lower access or binding to GSH [67]. It has been reported that DCA metabolism could be gender- and sex hormone dependent in rats: activity of glycolate oxidase in gonad-intact females and gonadectomized male rats was significantly lower than in gonad-intact males ones (glycolate oxidase is involved in the conversion of DCA metabolite glycolic acid to oxalate in rat liver); testosterone increases and estrogens decrease activity of glycolate oxidase in male rats [68]. The preclinical studies of DCA metabolism were conducted only in male rats [69–74]. Data on DCA metabolism in males cannot be directly extrapolated to females. The scientific guidelines for the preclinical safety evaluation of pharmaceuticals require both genders to be used [75]; the principles of Good Clinical Practice require that the available nonclinical information on an investigational product should be adequate to support the proposed clinical research [76]; e.g., gender-related investigational medicines effects remain to be explored firstly by preclinical studies.

## 5. Conclusions

- The DCA treatment decreases the thymus weight of gonad-intact rats of both genders, but such impact is absent in castrated rat groups, indicating the synergistic pharmacological mechanism of DCA and gonad hormones.
- The different mechanisms are responsible for changes in thymus epithelial cells transformation into Hassall's corpuscles under the DCA effect in gonad-intact and castrated animals.
- The DCA treatment decreases the NKCC1 gene RNA expression in the gonad-intact and castrated males, and no such effect was determined in females.
- The investigation of the DCA treatment efficacy should be evaluated with regard to gender and the exposition of gonad hormones.

## Figures and Tables

**Table 1:** The rat thymus weight data in male and female study groups.

Study group	Thymus weight (g)	
	mean (95 % CI)	
	males	females
Gonad-intact rats:	0.61 (0.47–0.78)	0.48 (0.42–0.56)
control		

DCA-treated	0.38 (0.33–0.43)^a^	0.32 (0.27–0.37)^b^

Castrated rats:	0.80 (0.69–0.92)^a^	0.85 (0.64–1.06)^b^
control		

DCA-treated	0.69 (0.58–0.80)	0.65 (0.46–0.84)

^a^ *p* – significant compared with the gonad-intact male control.

^b^ *p* – significant compared with the gonad-intact female control.

**Table 2:** The number ofHassall's corpuscles in thymus of the study groups.

Study group	Number of HCs per mm^2^	
	[median (range)]	
	males	females
Gonad-intact rats:	0.05 (0–0.16)	0.05 (0–0.26)
control		

DCA-treated	0.48 (0.18–1.0)^a^	0.2 (0.07–0.48)

Castrated rats:	0.26 (0.22–0.55)^b^	0.28 (0.16–0.49)^c^
control		

DCA-treated	0.4 (0.15–0.62)	0.26 (0.07–0.34)

^a,b^ *p* – significant compared with the male gonad-intact control group.

^c^ *p* – significant compared with the female gonad-intact control group.

**Table 3:** Correlation (*r*) among thymus weights, Hassall's corpuscles number per mm^2^, ∆CT, and Z test value (*z*) of the study groups.

Index	Rat group			
	gonad-intact		castrated	
	males	females	males	females
r between HCs and thymus weight in:				
control	0.55	0.15	0.03	0.49
DCA-treated	-0.94^a^	-0.49	0.83^c^	-0.30
*z*	*2.92* ^b^	*0.84*	*-1.42*	*0.93*

*r *between ∆CT and thymus weight in:				
control	-0.20	-0.50	-0.14	0.09
DCA-treated	-0.43	-0.60	-0.43	0.70
*z *	*0.31*	*0.18*	*0.39*	*-0.85*

^a^p = 0.005, in gonad-intact males.

^b^ *p* = 0.00, compared *r* of the gonad-intact control with *r* of the DCA-treated males.

^c^ *p* < 0.04, in castrated males.

**Table 4:** RNA expression of NKCC1 in thymus of the study groups.

Study group	CT average		∆CT	∆∆CT	2^-∆∆CT^
	*Glpdh*	*Slc12a2*			
*Gonad-intact female*:					
control	23.529	31.130	7.600	-2.558	5.890
DCA-treated	24.183	29.225	5.042		
*Gonad-intact male*:					
control	26.298	27.406	1.108	3.283	0.103
DCA-treated	23.710	28.101	4.391^a^		

*Castrated female*:					
control	22.675	30.115	7.440	-0.362	1.285
DCA-treated	21.791	28.869	7.079		
*Castrated male*:					
control	24.717	30.646	5.929	2.048	0.242
DCA-treated	20.912	28.889	7.977^b^		

^a^ *p* < 0.0001, DCA-treated gonad-intact males compared with their control.

^b^ *p* = 0.015, DCA-treated castrated male compared with their control.

## Data Availability

All data generated or analyzed during this study are included in this published article. The datasets used and/or analyzed during the current study are available from the corresponding author on reasonable request.
